# A Fatal Twist: Volvulus of the Small Intestine in a 46-Year-Old Woman

**DOI:** 10.1155/2015/391093

**Published:** 2015-11-03

**Authors:** Jared Klein, Kathryn Baxstrom, Stephen Donnelly, Patrick Feasel, Paul Koles

**Affiliations:** ^1^Department of Pediatrics, Virginia Commonwealth University Health System, Richmond, VA 23220, USA; ^2^Department of Internal Medicine, University of Minnesota Medical Center, Minneapolis, MN 55454, USA; ^3^Departments of Family Medicine and Emergency Medicine, Christiana Care, Wilmington, DE 19899, USA; ^4^Department of Pathology, Cleveland Clinic, Cleveland, OH 44195, USA; ^5^Department of Pathology, Wright State University, Dayton, OH 45435, USA

## Abstract

A 46-year-old woman presented to two emergency departments within 12 hours because of acute abdominal pain. Physical exam demonstrated tenderness and epigastric guarding. An ultrasound was interpreted as negative; she was discharged home. Later that evening, she was found dead. Postmortem exam revealed acute hemorrhagic necrosis of a segment of jejunum secondary to volvulus. Clinical clues suggesting presentations of small bowel volvulus are usually nonspecific; the diagnosis is typically confirmed at surgery. Her unremitting abdominal pain, persistent vomiting, and absolute neutrophilia were consistent with an acute process. The etiology of this volvulus was caused by an elastic fibrous band at the root of the jejunal mesentery. While congenital fibrous bands are rare in adults, this interpretation is favored for two reasons. First, the band was located 20 cm superior to postsurgical adhesions in the lower abdomen and pelvis. Second, there was no history of trauma or previous surgery involving the site of volvulus.

## 1. Introduction

Small bowel volvulus (SBV) is defined as torsion of a loop of small bowel about the axis of its mesentery, resulting in partial or complete obstruction. SBV is a rare cause of small bowel obstruction in Western countries, comprising 1–6% of cases [1]. However, it accounts for 20–35% of small bowel obstructions in Asia, Africa, and the Middle East. A precipitating factor may be the ingestion of a large amount of fiber after extended periods of fasting during the Ramadan festival [1–3]. SBV may be primary, without any underlying anatomic abnormalities or known predisposing factors. In adults, SBV is most often secondary to postsurgical adhesions, fibrous bands involving the mesentery, or congenital malrotation of the small bowel [4]. In patients with clinical evidence of small intestinal obstruction, the diagnosis of SBV may be suggested by abdominal multislice CT scan that demonstrates the “whirl sign” due to twisting of the small bowel, mesentery, and mesenteric vessels [1, 3, 5–8]. SBV may lead to ischemic necrosis of the bowel, underscoring the necessity of prompt diagnosis and surgical intervention [2, 4, 5, 7]. Mortality rates vary depending on time delay before surgical intervention, but overall mortality ranges from 10 to 38% [1–3, 9]. We present a case of a 46-year-old woman whose clinical evaluation did not lead to surgical intervention, resulting in death due to complications of small bowel infarction.

## 2. Case Summary

A 46-year-old African-American woman came to the emergency department because of acute lower abdominal pain of four-hours duration. She described the pain as sharp and severe. She was nauseated and had vomited yellow, nonbloody fluid at home. Her past medical history was significant for hypertension and past surgical history included hysterectomy (for leiomyomas), unilateral oophorectomy, and appendectomy. Physical exam showed a soft, nondistended, nontender abdomen with no masses or guarding. Vital signs were temperature 99.8°F, pulse 100/min, respirations 12/min, and blood pressure 130/90 mmHg. Laboratory studies, including AST, ALT, Alkaline phosphatase, lipase, and electrolytes, were within normal limits. Complete blood count showed WBC 10,300/*μ*L with 88% neutrophils but was otherwise within normal limits. The patient was given one liter of normal saline, hydromorphone, promethazine, and ondansetron. She was discharged 4 hours after arrival and told to follow up with her primary care physician and to return to the ED if symptoms worsened.

The patient was brought to another emergency department seven hours later with continuing abdominal pain that had begun 15 hours earlier. Vital signs were temperature 97.6°F, pulse 116/min, respirations 20/min, and blood pressure 140/90 mmHg. Physical exam showed positive rebound tenderness and guarding in the epigastrium. Bowel sounds were present, and the abdomen was not distended. Lipase, amylase, AST, ALT, alkaline phosphatase, and total bilirubin were again within normal limits. A right upper quadrant ultrasound was performed and interpreted as negative for gallbladder, common bile duct, or pancreatic pathology. The patient was treated with a liter of normal saline, morphine, and ondansetron. Her family asked for something “to calm her down”; she was given prochlorperazine and diphenhydramine. She was discharged four hours after arrival and told to call her doctor for a follow-up appointment. That evening at home, the patient spoke by phone with a relative who felt she was confused and not responding appropriately. When the relative arrived at the patient's home, the patient was unresponsive with bloody emesis on and around her body.

**Figure 1:**
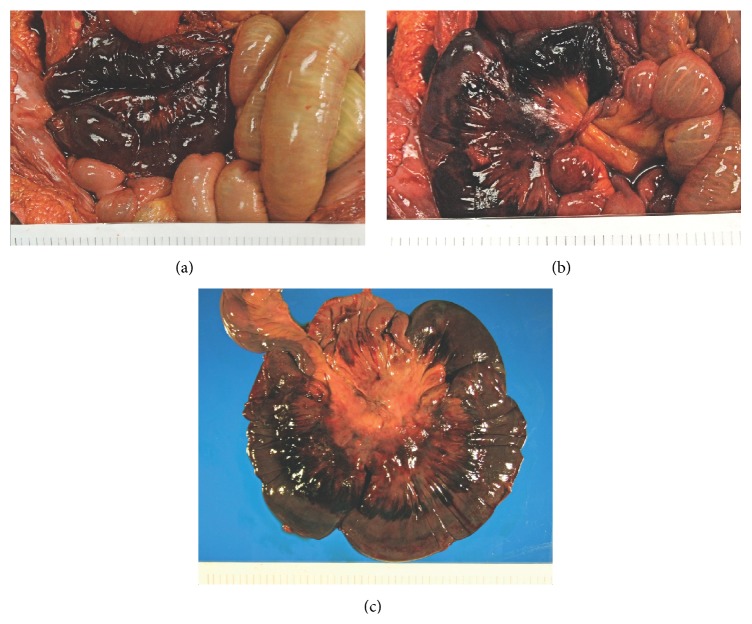
(a) Sixty-centimeter segment of necrotic jejunum secondary to volvulus. (b) Note the band of elastic tissue causing tissue strangulation and necrosis. (c) Necrotic segment of bowel with transmural necrosis.

**Figure 2:**
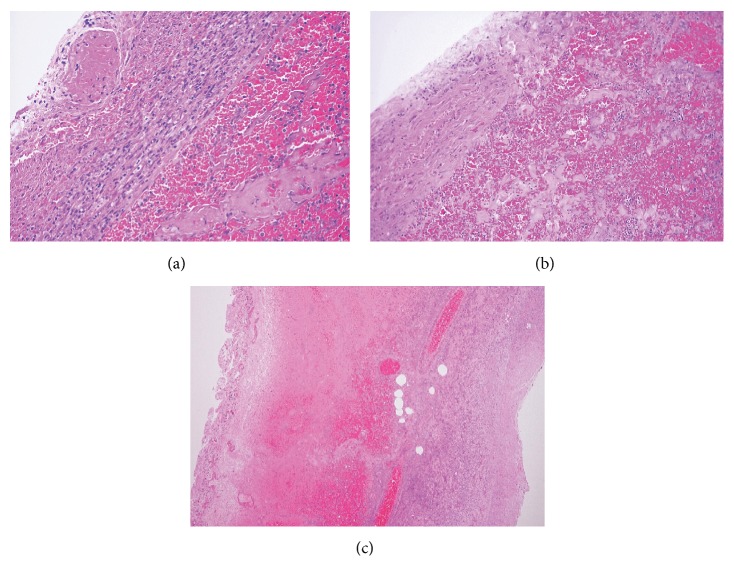
(a) Transmural vascular congestion and extensive hemorrhage. (b) Smooth muscle fibers in the muscularis propria split and fragmented, with strands of myocyte cytoplasm floating in extravasated blood. (c) Focally, only a thin layer of muscularis propria remains beneath the serosa.

Postmortem exam revealed acute hemorrhagic necrosis of a 60 cm long segment of jejunum secondary to volvulus (Figure 1(a)). The mesentery and necrotic segment were twisted and tethered under a thick band of elastic connective tissue in the posterior upper abdomen (Figure 1(b)). The band was located 10 cm inferior to the edge of the liver and 4 cm right of midline. Duodenum and jejunum proximal to this segment were dilated. Mesenteric arteries supplying the segment contained no thrombi. The necrotic segment showed diffuse thinning of the muscularis propria and transmural dark purple discoloration (Figure 1(c)). There were no masses, ulcers, scarring, or perforations. Small intestine distal to the volvulus was normal in color and contained serosanguineous fluid. Microscopically, the jejunum showed transmural vascular congestion and extensive hemorrhage (Figure 2(a)). The mucosa was mostly absent, showing only scattered remnants of villi with hypocellular lamina propria and no intact epithelium. Smooth muscle fibers in the muscularis propria were split and fragmented, with strands of myocyte cytoplasm floating in extravasated blood (Figure 2(b)). Focally, only a thin layer of muscularis propria remained beneath the serosa (Figure 2(c)).

## 3. Discussion

### 3.1. Pathogenesis

Volvulus is a special form of mechanical intestinal obstruction. It results from abnormal twisting of a loop of bowel around the axis of its own mesentery [5]. Volvulus can be primary, without any predisposing anatomic abnormalities and risk factors, or secondary to congenital or acquired lesions [2]. The mechanism of primary SBV has been correlated with the ingestion of a large amount of fiber-rich foods in a short time. The subsequent forceful small bowel peristalsis is believed to cause primary SBV [10]. Secondary causes are numerous and include postsurgical adhesions, malrotation, and, as in our case, congenital fibrous bands. Adhesions are the most common cause in adults; congenital fibrous bands are rare and typically cause symptomatic obstruction in children [11]. Mesenteric rotation (torsion) causes vascular insufficiency, and resultant ischemia and tissue hypoxia. Depending on the etiology, intestinal volvulus may present as a closed-loop obstruction in which a segment of bowel is occluded at two points along its length, resulting in fluid sequestration and gas production due to bacterial overgrowth. Substantial increases in intraluminal pressure and dilation of the bowel segment further compromise vascular supply to the intestinal wall, ultimately leading to hemorrhagic infarction and perforation [10]. In our case, the fibrous band acted as a point of strangulation resulting in the necrosis of the small bowel. The degree of circulatory impairment depends on the tightness of the twist; infarction occurs in approximately 50% of cases [11]. If an extensive segment of bowel is involved, large volumes of blood and plasma are extravasated into the intestinal wall and lumen [12]. Gut bacteria are introduced into the lymphatics and capillaries as mucosal integrity is lost, potentially leading to septic shock, multiorgan failure, and death.

### 3.2. Diagnosis

Patients with SBV may present with colicky abdominal pain, nausea, vomiting, abdominal distention, and obstipation [13]. However, as seen with this case, some of these symptoms may be blatantly present and others may be more subtle or absent entirely. In addition, certain physical exam findings such as tachycardia and rebound tenderness as well as abdominal radiography yield nonspecific results that do not differentiate this disease process from other causes of small bowel obstruction [1, 6]. The clinician must utilize multislice CT with contrast to achieve visualization of the underlying pathology [1]. Also, three-dimensional reconstruction of abdominal angiography can delineate the features of the mesenteric vessels [7]. It is uncertain why neither emergency department chose to use this imaging modality, which may have produced a better outcome. Typically, when a patient requires opioid management, further workup is initiated to identify the underlying etiology. Of note, CT scans may reveal torsion of loops of small bowel around the mesenteric vessels and mesentery known as the “whirl sign” [1, 7]. The sensitivity and specificity of the whirl sign in the diagnosis of SBV are variable; while not pathognomonic, it remains a useful finding [4, 8, 14]. Other radiographic signs, such as the “spoke wheel,” “beak,” and “barber pole” signs, have been described in the literature as well [6, 15]. In addition, Sandhu et al. demonstrated that multiple transition points, defined as a segment of dilated bowel followed by a segment of collapsed bowel located in the posterior abdomen, are more prevalent in patients with SBV compared to other etiologies of small bowel obstruction [14].

### 3.3. Treatment

Emergent surgical intervention is necessary to avoid ischemic necrosis or perforation of the bowel [1, 5]. Exploratory laparotomy can be performed to confirm the diagnosis and guide further decision-making [16]. According to Grasso et al., there have not been any prospective, randomized trials comparing outcomes of derotation versus resection with anastomosis. Most authors agree that resection is required for necrotic bowel [1–5, 9]. In the absence of necrosis, if the bowel appears to be edematous or congested, simple derotation, with or without fixation of the involved small bowel, may be considered. However, this procedure is associated with recurrence of SBV [9]. Published mortality rates vary, but the consensus is 10–35% [1–3, 9]. Patient-specific factors such as age, comorbidities, and general health play a role in the decision of which treatment option to pursue [3]. Immediate surgical intervention is highly encouraged to prevent adverse outcomes including peritonitis, sepsis, and death [2].

### 3.4. Summary

In summary, clinical clues to the diagnosis of SBV are often nonspecific, which is why the clinician must always consider the differential diagnosis of SBV in cases of acute abdominal pain. Often, abdominal pain will precede alterations in laboratory blood work results by hours. In this case, causes for concern included the history of unremitting abdominal pain for at least 15 hours, vomiting, the abdominal exam findings, and an absolute neutrophilia on presentation to the first emergency department. When evaluating acute abdominal pain, a CT scan may be more useful than ultrasound in providing evidence of etiology or anatomic localization. As stated previously, this is the key imaging modality that may demonstrate the “whirl sign” that can suggest the diagnosis of SBV. Surgical exploration is indispensable to confirm the diagnosis of SBV and prevent excess morbidity or mortality as was the result with our patient. In our case, the fibrous band causing volvulus was located at the mesenteric root of the ischemic segment of jejunum. While a congenital band is rare in adults, we favor this interpretation of the etiology of volvulus in our patient for two reasons. First, the band was anatomically isolated, located on a significant distance (about 20 cm) from the mild postsurgical fibrous adhesions identified in the lower abdomen and pelvis. Second, the band's large size is difficult to explain as an acquired lesion, especially without a history of trauma or previous surgery in the epigastric region. In conclusion, it was the lack of proper imaging that prevented the diagnosis of SBV in our patient, which resulted in her death. If a CT scan had been performed, she may have undergone surgery for small bowel detorsion or resection, which could have saved her life.
